# Iterated local search algorithm for solving the orienteering problem with soft time windows

**DOI:** 10.1186/s40064-016-3440-6

**Published:** 2016-10-12

**Authors:** Brahim Aghezzaf, Hassan El Fahim

**Affiliations:** Laboratoire Informatique et Aide à la Décision (LIAD), Département de Mathématiques et Informatique, Faculté des Sciences Aïn Chock, Université Hassan II de Casablanca, Km 8 Route d’El Jadida, 5366 Maarif, 20100 Casablanca, Morocco

**Keywords:** Combinatorial optimization, Orienteering problem, Soft time window, Iterated local search, Variable neighborhood search

## Abstract

In this paper we study the orienteering problem with time windows (OPTW) and the impact of relaxing the time windows on the profit collected by the vehicle. The way of relaxing time windows adopted in the orienteering problem with soft time windows (OPSTW) that we study in this research is a late service relaxation that allows linearly penalized late services to customers. We solve this problem heuristically by considering a hybrid iterated local search. The results of the computational study show that the proposed approach is able to achieve promising solutions on the OPTW test instances available in the literature, one new best solution is found. On the newly generated test instances of the OPSTW, the results show that the profit collected by the OPSTW is better than the profit collected by the OPTW.

## Background

In orienteering problem (OP) a set of potential customers is given; the service for these customers is optional during the current planning time horizon since the travel cost of the route is limited. The travel cost is often expressed as the travel time or the travel distance. Thus, a positive value called profit is associated with every customer making its visit more or less attractive. The name of this routing problem originates from a game in which competitors have to visit a set of control points in a given area. If the control point is visited, the competitor gets a profit. The winner of the game is the competitor who collects maximum profits and gets to the end point within a prescribed amount of time. As a routing problem, the OP consists in finding the route visiting a subset of customers that maximizes the total collected profit while satisfying the maximum duration constraint. The OP is also known in the literature as the Selective Traveling Salesman Problem (Thomadsen and Stidsen 2003), the Maximum Collection Problem (Butt and Cavalier 1994) and the Bank Robber Problem (Awerbuch et al. 1998).

Few vehicle routing problems have such applicability as OP. This problem arises in a variety of applications including design of tourist trips to maximize the value of the visited attractions (Vansteenwegen and Oudheusden 2007), recruiting of athletes from high schools for a college team (Butt and Cavalier 1994), delivery of home heating fuel where the urgency of a customer for fuel is treated as a profit (Golden et al. 1984), routing of oil tankers to serve ships at different locations (Golden et al. 1987) and reverse logistics problem of a firm that aims to collect used products from its dealers (Aras et al. 2011).

The OP is a well-studied combinatorial optimization problem that was first presented and heuristically solved by Tsiligirides (1984). Several heuristics and metaheuristics were proposed for the solution of the OP [the reader is referred for example to the papers by Tasgetiren (2001), Ramesh and Brown (1991) and Gendreau et al. (1998)].

In the time windows version of the OP called the orienteering problem with time windows (OPTW), customers have hard time windows and service times. In hard time windows, arriving at customer later than latest time of its time window is strictly forbidden. A waiting is incurred if the vehicle reaches to a customer before its earliest time window. In OPTW, the objective is designing the route that maximizes the total collected profit while satisfying the time limit duration and the hard time windows constraints.

In recent years there has been considerable interest for the OPTW which has led to a significant body of literature. The authors in Righini and Salani (2009) proposed a bidirectional dynamic programming algorithm for solving the OPTW to optimality. They use a technique named decremental state space relaxation in which the dynamic programming algorithm takes advantage of a state space relaxation. The authors in Duque et al. (2015) proposed an exact algorithm based on pulse framework for solving the OPTW to optimality. Most studies have focused on designing heuristic algorithms, several heuristics and metaheuristics were then proposed for the solution of the OPTW [the reader is referred for example to the papers by Vansteenwegen et al. (2009), Lin and Yu (2012), Labadie et al. (2011, 2012), Montemanni and Gambardella (2009), Tunchan (2014), Gunawan et al. (2015) and Lahyani et al. (2016)]. For a recent survey on OP and OPTW the reader is referred to the paper by Gunawan et al. (2016).

Problem related to the problem studied in this research is the vehicle routing problem with soft time windows (VRPSTW). In many real-life problems, some or all customers’ time windows are not so strict that can be violated with appropriate penalties. Such kind of time constraint is called soft time window. In VRPSTW, vehicles are allowed to serve customers before the earliest and/or after the latest time windows bounds. This type of time windows is useful for the dispatcher when:

- The number of routes needed for hard time windows exceeds the number of available vehicles.
- A study of cost-service tradeoffs is required.
- The dispatcher has qualitative information regarding the relative importance of hard time windows across customers.

Besides, relaxing time windows can result in lower total costs without hurting customers’ satisfaction significantly. In the literature, there are different ways of relaxing time windows which lead to different variants of VRPSTW.

- If a vehicle arrives before the earliest bound of the time window, it waits as in hard time windows case. However, late service is allowed if an appropriate penalty is paid. The authors in Taillard et al. (1997) proposed a tabu search heuristic for this variant.
- Both early and late services are allowed by paying appropriate penalties. The authors in Koskosidis and Solomon (1992) proposed an optimization-based heuristic for this variant.
- Both early and late services are allowed as in the second variant. However, the maximum allowable violation of the time windows and the maximum waiting time allowed are limited. This is the variant studied by Chiang and Russell (2004) and Balakrishnan (1993). The authors in Balakrishnan (1993) described three heuristics for solving this variant. While the authors in Chiang and Russell (2004) proposed a tabu search heuristic to deal with this variant.

For more detail about these relaxation schemes and the algorithms proposed in the literature to solving them, the reader is referred to the paper by Vidal et al. (2015).

### Contributions

We observed when solving orienteering problems with time windows as in Aghezzaf and Fahim (2015) that the gap between the total travel time of a route and the travel time limit is significant especially on instances with long scheduling horizon. Thus, we have decided to manage this gap by allowing relaxation of time windows in order to improve the profit collected by the vehicle. Furthermore, there are many practical reasons for allowing violation of time windows:

- Many applications do not require hard time windows.
- In many cases travel times cannot be accurately known.
- Customers may be unwilling to set precise time windows in advance and simply prefer the flexibility to alter their delivery requests.

The contribution of this paper is twofold:

- We introduce and define a new routing denoted the orienteering problem with soft time windows (OPSTW). To the best of our knowledge, this is the first study dealing with orienteering problem with soft time windows. In this routing problem, late service is allowed if an appropriate penalty is paid. In this relaxation scheme, we are placing restrictions on both the penalty payable and the waiting time. We think that OPSTW solutions can result in routes visiting a significant number of potential customers without hurting customers’ satisfaction significantly. Furthermore, soft time windows can provide a workable plan of action for decision makers when hard time windows are not required or when it is not possible to visit all customers during the current planning time horizon which is the case for the OPTW.
- We develop a hybrid algorithm that combines an iterated local search with a variable neighborhood search for this specific problem. We also apply it to standard instances and compare its performance to that of other algorithms proposed in the literature for the OPTW.

The rest of this paper includes four additional sections. “Mathematical model” section defines the mathematical notation and formulation of OPSTW. “Solution algorithm” section describes the proposed hybrid algorithm. “Computational results” section presents the computational results and compares our algorithm against published results both with regard to solution quality and computational time. The last section is devoted to the conclusions.

## Mathematical model

The OPTW studied in this paper can be described as follows : let $G=(V,E)$ be a complete graph, where $V=\{0,1,\ldots ,n\}$ is a vertex set and $E=\{(i,j) \in V^{2}\, i \ne j\}$ is an arc set. Vertex 0 denotes a depot at which the vehicle starts and ends its tour. The set of vertices $C=\{1,\ldots , n\}$ specify the location of a set of *n* customers. Each vertex $i \in V$ has an associated profit $p_i$ ($p_0 = 0$), a service time $S_i$ ($S_0 = 0$) and a time window $[e_i, l_i]$ which is assumed to be hard. Each arc $(i, j) \in E$ has an associated cost $t_{ij}$ which represents the time required to travel from vertex *i* to vertex *j*. The cost $t_{ij}$ is defined as the Euclidean distance between the points corresponding to *i* and *j*. The arrival time to a customer *i* is denoted $a_i$; the beginning of service time is denoted $b_i$. The objective is to design a route *R* that maximizes the total collected profit subject to the following:

- The route *R* cannot start before $e_0$ and cannot end after $l_0$.
- The service to a customer *i* cannot start before $e_i$ and if the vehicle arrives too early it can wait for a certain period of time $w_i = max(e_i - a_i, 0)$ and serves that customer.
- Every customer is visited at most once.
- The total travel time of *R* is limited by a time limit $T_{max}$.

In order to formulate the model we define the following decision variables :

- $x_{ij}$ binary variable equal to 1 if the vehicle travels directly from vertex *i* to vertex *j*, and 0 otherwise.
- $y_i$ binary variable equal to 1 if vertex *i* is visited, and 0 otherwise.
- $b_i$ beginning of service time at customer *i*.

M is a large value.

The OPTW can be formulated as the following mixed integer linear programming model:

1 $$\begin{aligned}&\text {max}\,\,f = \sum _{i \in C} {p_{i} y_{i}} \end{aligned}$$

2 $$\begin{aligned}&\text {subject to:} \\&\sum _{j \in C} {x_{0j}} = \sum _{i \in C} {x_{i0}} = 1 \end{aligned}$$

3 $$\begin{aligned}\sum _{i \in V} {x_{il}} = \sum _{j \in V} {x_{lj}} \le 1 \quad \forall l \in C\end{aligned}$$

4 $$\begin{aligned}b_{i} + S_{i} + t_{ij}- b_{j} \le M(1 - x_{ij}) \quad\forall i,j \in V \end{aligned}$$

5 $$\begin{aligned}\sum _{i \in V} \left(S_{i}y_{i} + \sum _{j \in V} {t_{ij} x_{ij}}\right) \le {T_{max}} \end{aligned}$$

6 $$\begin{aligned}e_{i} \le b_{i} \le l_{i} \quad\forall i \in V \end{aligned}$$

7 $$\begin{aligned}x_{ij}, y_{i} \in \{0,1\} \quad \forall i,j \in V \end{aligned}$$

8 $$\begin{aligned}b_{i} \in {{\mathbb {R}}}^{+}\cap [e_0, l_0] \quad\forall i \in V \end{aligned}$$

The objective function (1) maximizes the total collected profit. Constraint (2) guarantees that the route starts and ends at vertex 0 (depot). Constraints (3) and (4) determine the connectivity and the time line of the route. Constraint (5) ensures the maximum time duration constraint of the route. Constraints (6) restrict the start of the visit to the time windows. Constraints (7) and (8) are variables definition.

In OPSTW, the time window of every customer $i \in C$ can be enlarged to an outer time window $[e_{i},l_{i} + P_{max}] = [e_i, \hat{l}_{i}]$, where $P_{max}$ is an upper bound on the maximum allowable time window violation. An appropriate penalty $P^{penalty}_{i}$ is then paid if the service starts late that is $a_{i} \in ]l_{i}, \hat{l}_{i}]$. The penalty function can be defined as follows:

9 $$\begin{aligned} P^{penalty}_{i} ={\left\{ \begin{array}{ll} 0 & \quad {\text{if}}\; e_{i} - W_{max} \le a_{i} \le l_{i} \\ a_{i} - l_{i} & \quad {\text {if}} \; l_{i} < a_{i} \le \hat{l}_{i} \end{array}\right. } \end{aligned}$$

One can express the OPSTW objective function as a combination between the total collected profit (the classic objective for OPTW) and the total penalty for time windows violations. In our formulation, we do not express it that way since this will change the nature of the faced problem and the aim of this work. In our OPSTW formulation, the total penalty and the total waiting time are expressed as travel costs and added to the total travel time of the route. Then, constraint (5) of the previous model changes as follows:

10 $$\begin{aligned} \sum _{i \in V} \left(\left(S_{i} + P^{penalty}_{i} + w_{i}\right) y_{i} + \sum _{j \in V} {t_{ij} x_{ij}}\right) \le {T_{max}} \end{aligned}$$

Since in our formulation we take into account the fact that $w_i \le W_{max}$ for each routed customer *i*, the following constraint is added to the model.

11 $$\begin{aligned} (e_j - (b_i + S_i + t_{ij}))x_{ij}\le (W_{max})x_{ij} \quad \forall i,j \in V \end{aligned}$$

Regarding the time windows constraints they change as follows:

12 $$\begin{aligned} e_{i}\le b_{i} \quad \forall i \in V \end{aligned}$$

13 $$\begin{aligned} b_{0}\le l_{0}\end{aligned}$$

14 $$\begin{aligned} b_{i}\le \hat{l}_{i} \quad \forall i \in C \end{aligned}$$

In the next subsection, we will describe the approach that we propose to deal with the OPSTW.

## Solution algorithm

At its core, the approach proposed to solve the OPSTW is an iterated local search (ILS). ILS is a local search based metaheuristic that was introduced in Lourenço et al. (2003) to solve combinatorial optimization problems. Let *S* be the starting solution for the ILS process. At each iteration, a diversification phase is firstly applied by perturbing *S*. An intensification phase is then performed around the perturbation output by applying a local search procedure to produce a solution $\acute{S}$. If $\acute{S}$ satisfies an acceptance criterion, it replaces the starting solution and the next perturbation phase is performed from that solution. Otherwise, $\acute{S}$ is discarded and the search returns to the previous starting solution. In the proposed ILS algorithm, a variable neighborhood search (VNS) is applied to $\acute{S}$ even if it is better than *S* or not. The objective is intensifying the search around $\acute{S}$, which is a local optima with respect to the local search procedure, in order to explore promising regions of the solution space. Algorithm 1 illustrates the steps of the proposed hybrid ILS (HILS) algorithm. The stopping condition used is the maximum number of iterations allowed $L_{max}$. In the next subsections, we will describe the components of the proposed ILS algorithm which are the initial solution procedure, the perturbation operator, the local search procedure and the variable neighborhood search. 
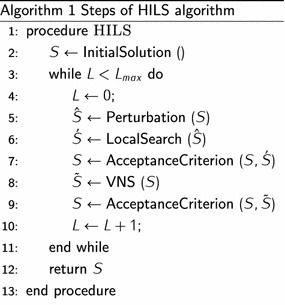


### Initial solution

We propose three insertion heuristics to generate a set of solutions and we pick the best as the starting solution for the ILS process. These heuristics follow the scheme of the insertion heuristic proposed by Solomon (1987) for the vehicle routing problem with time windows (VRPTW), they differ in the expression of the criterion used to compute the best feasible insertion place of each unrouted customer on the current partial route. In the following subsections we will first present the procedure implemented to check the feasibility of an insertion and then we will present the initial solution procedure.

#### Feasibility check

Let $R = (i_0, i_1, i_2, \ldots , i_{m-1},i_m), i_0 = i_m = 0$ be the current partial route and let *u* be an unrouted customer. We define a feasible place of the customer *u* in *R* as a position $(i_{p-1}, i_p) \, p \in \{ 1, \ldots , m\}$ in *R* for which, if *u* is inserted between the adjacent vertices $i_{p-1}$ and $i_p$ then:

The waiting time at customer *u* given by Eq. (15) satisfies the following:

15 $$\begin{aligned} w_{u} = max(0, e_{u} - (b_{i_{p-1}} + S_{i_{p-1}} + t_{i_{p-1}u})) \le W_{max} \end{aligned}$$

The time duration constraint on the route is still satisfied, that is:

16 $$\begin{aligned}\sum_{r=1}^{p-1}t_{i_{r-1}i_{r}} + \sum _{r=1}^{p-1}\left(w_{i_{r}} + S_{i_{r}} + P^{penalty}_{i_{r}}\right) + t_{i_{p-1} u} + t_{u i_{p}} - t_{i_{p-1}i_{p}} + S_{u} + w_{u} + P^{penalty}_{u} + \sum _{r=p+1}^{m}t_{i_{r-1}i_{r}} + \sum _{r=p}^{m-1} \left(w_{i_{r}} + S_{i_{r}} + P^{penalty}_{i_{r}} \right) \le T_{max} \end{aligned}$$

All vertices subsequent to the inserted customer *u* still satisfy at most their outer time windows. This is done using the conditions for time feasibility proposed by Solomon (1987) as follows:

17 $$\begin{aligned} b_{u} \le \hat{l_{u}}, \quad {\text{and}} \quad b_{i_{r}} + PF_{i_{r}} \le \hat{l_{i_{r}}}, \quad p \le r \le m \end{aligned}$$

where:

18 $$\begin{aligned} PF_{i_{p}} = b^{new}_{i_{p}} - b_{i_{p}} \end{aligned}$$

The metric $b^{new}_{i_{p}}$ corresponds to the beginning of service at customer $i_{p}$ (which is the arrival time at the depot if $p =m$) given that customer *u* is inserted between $i_{p-1}$ and $i_{p}$. This metric is computed as follows:

19 $$\begin{aligned} b^{new}_{i_{p}} = max(e_{i_{p}}, b_u + S_u + t_{u i_{p}}) \end{aligned}$$

It is clear that $PF_{i_{p}} \ge 0$ since the matrix $(t_{ij})_{(i, j)\in E}$ satisfies the triangle inequality. This metric is computed for the rest of the subsequent vertices as follows:

20 $$\begin{aligned} PF_{i_{r+1}} = max\{0, PF_{i_{r}} - w_{i_{r+1}}\}, \quad p \le r \le m-1 \end{aligned}$$

#### Insertion heuristics

Each insertion heuristic $H_l$, $l \in \{1,2,3\}$ starts by determining the best feasible place of each unrouted customer on *R*. Such position is defined as the position (*i*(*u*), *j*(*u*)) for which:

21 $$\begin{aligned} C_{1}(i(u), u, j(u)) = min[C^{H_{l}}_{1}(i_{p-1}, u, i_{p})], \quad p \in \{1, 2, \ldots , m\} \end{aligned}$$

The first insertion heuristic $H_{1}$ computes this position using the following criterion:

22 $$\begin{aligned} C^{H_{1}}_{1}(i_{p-1}, u, i_{p}) = \alpha _{1}(t_{i_{p-1} u} + t_{u i_{p}} - t_{i_{p-1}i_{p}} + \alpha _{3}S_u) + \alpha _{2}(b^{new}_{i_{p}} - b_{i_{p}}) \end{aligned}$$

The second insertion heuristic $H_{2}$ computes this position using the following criterion:

23 $$\begin{aligned} C^{H_{2}}_{1}(i_{p-1}, u, i_{p}) = \alpha _{1}(t_{i_{p-1} u} + t_{u i_{p}} - t_{i_{p-1}i_{p}}+\alpha _{3}S_u) + \alpha _{2}w_{u} \end{aligned}$$

While the third insertion heuristic $H_{3}$ computes this position using the following criterion:

24 $$\begin{aligned} C^{H_{3}}_{1}(i_{p-1}, u, i_{p}) = \alpha _{1}(t_{i_{p-1} u} + t_{u i_{p}} - t_{i_{p-1}i_{p}}+\alpha _{3}S_u) + \alpha _{2}\theta _{u} \end{aligned}$$

The metric $\theta _{u}$ corresponds to the time difference between the completion of service at customer $i_{p-1}$ (which is the departure time from the depot if $p=1$) and the beginning of service at customer *u*. This metric is expressed as follows:

25 $$\begin{aligned} \theta _u = b_u - (b_{i_{p-1}} + S_{i_{p-1}}) \end{aligned}$$

The weights $\alpha _1$, $\alpha _2$ and $\alpha _3$ define the relative contribution of each individual metric in the computing of the best feasible insertion place. The parameter $\alpha _1$ takes into account the saving in travel time by inserting *u* between $i_{p-1}$ and $i_{p}$.

Then, each insertion heuristic selects the best unrouted customer *v* according to the criterion given by Eq. (26) and inserts it in the current partial route *R*.

26 $$\begin{aligned} C_{2}(i(v), v, j(v)) = \min _{u \in \nabla } [C_{2}(i(u), u, j(u))] \end{aligned}$$

where $\nabla$ is the set of unrouted customers having at least one feasible place on the current partial route. The criterion $C_{2}(i(u), u, j(u))$ is expressed as follows:

27 $$\begin{aligned} C_{2}(i(u), u, j(u)) = \dfrac{C^{H_{l}}_{1}(i(u), u, j(u))}{p^{\alpha _{4}}_{u}} \end{aligned}$$

The parameter $\alpha _4$ is the exponent of the profit of customer *u*. The procedure of customer insertion is repeated, for each insertion heuristic, until no further unrouted customer can be inserted into *R*. The insertion procedure terminates by providing the set of assigned customers and the sequence in which these customers are routed. This is repeated for a number of values for $\alpha _{i}, i \in \{1,2,3,4\}$ and the best overall solution is returned at the end. We compare two solutions using the following criteria in decreasing order: collected profit and time duration. Note that $\alpha _{i}, i \in \{1,2,3,4\}$ are positive weights that satisfy: $\alpha _{i} \ge 0, i \in \{1,2,3,4\}$ and $\alpha _1 + \alpha _2 = 1$.

### Perturbation

The perturbation operator used in the proposed ILS algorithm performs, around a solution, by selecting the customer with minimal profit and removing it from this solution. Note that after a removal, all vertices (customers + depot) following removed one are shifted towards the beginning of the route in order to ensure its continuity. As one can intuitively expect, this forward move can result in an infeasible solution since the waiting time on some customers may exceed the maximum allowable waiting time $W_{max}$. This is solved using local search procedure that will be described in the next subsection.

### Local search

The local search procedure tries to fulfill the available room in the solution, obtained through perturbation, by inserting other feasible unrouted customers.

On one hand and as one can intuitively expect, evaluating the possible insertion of each unrouted customer using the criterion presented in the initial solution procedure will increase the risk of inserting the set of customers just removed, and getting easily stuck in the initial solution. On the other hand, the solution obtained through perturbation can be infeasible.

Given a starting solution *S*, the solution obtained through perturbation is denoted $\hat{S}$. The local-search procedure performs, around $\hat{S}$, by inserting each unrouted customer in its first feasible place. Using this local search procedure, following effects are observed:

- an unrouted customer in *S* can be part of $\hat{S}$.
- a routed customer in *S* cannot be inserted in $\hat{S}$.
- the position of a customer in *S* can be changed to another position in $\hat{S}$.
- the customers of $\hat{S}$ are shifted towards the end of the route in order to avoid unnecessary waiting time. As a consequence, the solution obtained after local search procedure is feasible.

This procedure will help to re-optimize a solution, make it feasible if it is not and insert other feasible unrouted customers in order to improve the value of the incumbent solution.

### Variable neighborhood search

Variable neighborhood search (VNS) is a local search based metaheuristic that was introduced in Mladenović and Hansen (1997) for solving combinatorial optimization problems. The basic idea behind this metaheuristic is a systematic change of neighborhoods both in descent phase using a local search procedure, and in shaking phase using a set of neighborhood structures. VNS is a stochastic algorithm in which, first, a finite set of neighborhood structures is defined. We denote by *N*(*S*) the set of solutions in the neighborhood of *S*. Each VNS iteration is composed of three steps: shaking, local search and move. At each iteration, a solution $\acute{S}$ is randomly generated from the neighborhood of *S*. A local search is then applied with $\acute{S}$ as the initial solution, the obtained solution is denoted $\hat{S}$. If $\hat{S}$ is better than *S*, the search moves to $\hat{S}$ and continues by considering the first neighborhood structure. Otherwise, *k* is incremented. The steps of the proposed VNS algorithm are given by Algorithm 2. Before defining the neighborhood structures used in the developed VNS, we first define two metrics $\varphi _{S}, \vartheta _{S} \in {{\mathbb {N}}}$. To do that, let us denote with $S=(0,c_1, c_2, \ldots , c_m, 0)\, c_h \in C, h \in \{1,\ldots ,m\}$ a feasible solution. These metrics are defined as follows: 
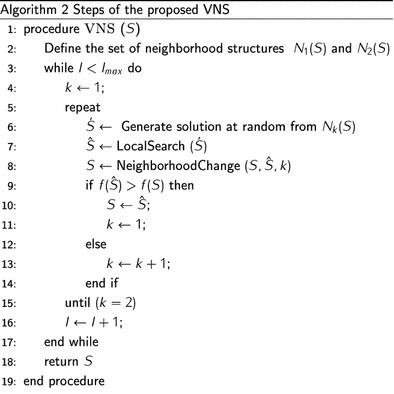

28 $$\begin{aligned} \varphi _{S}= \left\lfloor \dfrac{m}{2} \right\rfloor \end{aligned}$$

29 $$\begin{aligned} \vartheta _{S}= \left\lceil \dfrac{m}{2} \right\rceil \end{aligned}$$

The first neighborhood of *S* denoted $N_1(S)$ is defined as the set of all solutions obtained by removing two customers, one from the set $(c_1, c_2, \ldots , c_{\varphi _{S}})$ and the other from the set $(c_{\vartheta _{S}}, c_{\vartheta _{S}+1}, \ldots , c_m)$.

The second neighborhood $N_{2}(S)$ is defined as the set of all solutions obtained by removing a set of customers $(c_{k}: k \in [1,j])$ such that $j \in [1,m]$. The idea behind using floor and ceiling functions to define these neighborhood structures is to reduce the size of the neighborhoods and then the computational time.

## Computational results

The proposed algorithm is coded in Java; the experiments are performed on a personal computer Intel(R) with 2.1 GHz and 4 GB of RAM. We test, first, our algorithm on OPTW test instances. Based on these instances, we design test instances for the OPSTW. Each experiment is performed, on each test instance, five times for which the average results are presented. The presented computational times are the average times over five runs.

### Test instances

The authors in Righini and Salani (2009) designed test instances for OPTW based on 29 VRPTW test instances of Solomon (1987) namely C100, R100 and RC100, and on 10 Multi-depot Vehicle Routing Problem (MDVRP) test instances of Cordeau Cordeau et al. (1997) PR01-10. The number of customers for Cordeau instances varies between 48 and 288 customers; while all Solomon instances have 100 customers. The author in Vansteenwegen (2008) designed OPTW test instances based on Solomon instances by considering the first 50 customers $(n = 50)$. The authors in Montemanni and Gambardella (2009) designed other OPTW test instances based on 27 VRPTW test instances of Solomon namely, C200, R200 and RC200, and on 10 MDVRP test instances of Cordeau PR11-20. We design OPSTW test instances based on OPTW test instance namely PR01 by considering different values of $W_{max}$ and $P_{max}$. We set the maximum allowable waiting time to $W_{max} \in [5, 7]$, in increments of 0.5 %, of the maximum time duration $T_{max}$. While we set the maximum allowable time window violation to $P_{max} \in [1, 5]$, in increments of 0.5 %, of $T_{max}$. In all OPTW and OPSTW test instances, it is assumed that the maximum time duration $T_{max}$ is equal to $T_{max}=l_0 - e_0$.

### Parameter tuning

Preliminary experiments on OPTW test instances are conducted to set the values of $\alpha _{i}, i \in \{1,2,3,4\}$. The following values are tested: $\alpha _{i} \in [0,1], i \in \{1,2,3\}$ in increments of 0.1 units and $\alpha _{4} \in \{1,2,3,4\}$. The results indicate that given more importance to the metric related to the saving in travel time in the selection criterion has good influence on solution quality. Thus, the following values are selected:

- $$\alpha _1 \in \{ 0.9, 0.7, 0.5\}$$
- $$\alpha _2 \in \{0.1, 0.3, 0.5\}$$
- $$\alpha _3 \in \{0.1, 0.3, 0.5, 0.7, 0.9\}$$
- $$\alpha _4 \in \{2, 3, 4\}$$.

The proposed HILS algorithm has two parameters: a maximum number of iterations allowed $L_{max}$ which is ILS stopping condition, and $I_{max}$ the maximum number of iterations allowed in VNS process. The values of these parameters are determined performing several experiments on a subset of OPSTW test instances that was randomly selected. We test combinations of the following values during the experiments: $L_{max} \in \{ 20, 50, 100\}; I_{max} \in \{10, 20, 50\}$. The results of these experiments show that by increasing these parameters better results can be obtained at the expense of extra computational time. In this paper we are looking for a fast algorithm. Thus, we set these parameters to $L_{max} = 20$ and $I_{max} = 10$.

### Computational results on OPTW instances

In this subsection, we compare the results of HILS algorithm with the following state-of-the-art algorithms:

- I3CH: the iterative three-component algorithm of Hu and Lim (2014).
- IterLS: the iterated local search algorithm of Vansteenwegen et al. (2009).
- VNS: the variable neighborhood search algorithm of Tricoire et al. (2010).
- GVNS: the LP-based granular variable neighborhood search algorithm of Labadie et al. (2012).
- SSA: the slow version of the simulated annealing algorithm of Lin and Yu (2012).
- FSA: the fast version of the simulated annealing algorithm of Lin and Yu (2012).
- ILS: the iterated local search algorithm of Gunawan et al. (2015).
- GRASP-ELS: the hybrid algorithm of Labadie et al. (2011) that combines greedy randomized adaptive search procedure with evolutionary local search.
- ABC: the artificial bee colony algorithm of Tunchan (2014).
- DABC: the discrete artificial bee colony algorithm of Karabulut and Tasgetiren (2013).
- GA: the genetic algorithm of Karbowska-Chilinska and Zabielski (2014).
- ACS: the ant colony system algorithm of Montemanni and Gambardella (2009).
- EACS: the enhanced ant colony system algorithm of Montemanni et al. (2011).
- VAN: the iterated local search algorithm of Vansteenwegen (2008).

The computational results are given in Table 1. Column Instance-n gives the instance over which the algorithms are tested and the associated number of customers. Column BKS presents the latest best known solution as described in (http://centres.smu.edu.sg/larc/orienteering-problem-library). Column I3CH presents the profit obtained by I3CH algorithm (Profit), the percentual gap with the best known profit [Gap (%)] and the computational time required by I3CH algorithm on each run [CPU (s)]. Column ABC presents the average profit, over five runs, obtained by ABC algorithm, the percentual gap with the best known profit and the average computational time of five runs. Column VNS presents the average profit as described in Tricoire et al. (2013), over ten runs, obtained by VNS algorithm, the percentual gap with the best known profit and the average computational time of ten runs. Column ILS presents the profit obtained, over ten runs, by ILS algorithm, the percentual gap with the best known profit and the average computational time of ten runs. Column ACS presents the profit obtained, over five runs, by ACS algorithm, the percentual gap with the best known profit and the average computational time of five runs. Column GVNS presents the profit obtained, over five runs, by GVNS algorithm, the percentual gap with the best known profit and the average computational time of five runs. Column GRASP-ELS presents the profit obtained, over five runs, by GRASP-ELS algorithm, the percentual gap with the best known profit and the average computational time of five runs. Column SSA presents the profit obtained by SSA algorithm, the percentual gap with the best known profit and the computational time of one run. Column IterLS presents the profit obtained by IterLS algorithm, the percentual gap with the best known profit and the computational time of one run. Column FSA presents the profit obtained by FSA algorithm, the percentual gap with the best known profit and the computational time of one run. Column DABC presents the profit obtained, over five runs, by DABC algorithm, the percentual gap with the best known profit and the average computational time of five runs. Column HILS presents the profit obtained, over five runs, by our HILS algorithm, the percentual gap with the best known profit and the average computational time of five runs. Column GA presents the profit obtained, over sixteen runs, by GA algorithm, the percentual gap with the best known profit and the average computational time of sixteen runs.

Column Opt presents the optimal profit as described in Tricoire et al. (2010) and the associated computational time. Column VAN presents the profit obtained by VAN algorithm and the associated computational time. Column VNS presents the worst, best and average profit, over ten runs, of VNS algorithm and the average computational time. Column HILS presents the worst, best and average profit, over five runs, of HILS algorithm and the average computational time.

To ensure fair comparisons, the results of each algorithm are compared with the best known solutions with the computational times adjusted to the speed of the computers used to achieve these results. We summarize the experimental environment of each algorithm and compare their CPU speed in Table 2. As all algorithms are single threaded, we compare their CPU speed using the Super pi benchmark (http://www.superpi.net/). In Table 2, the Super pi column reports the number of seconds it takes each processor to compute the first one million digits of $\pi$. The processor used for IterLS algorithm is approximately two times faster than that used for GRASP-ELS and GVNS algorithms; the processor used for VAN algorithm is slower than that used for GRASP-ELS and GVNS algorithms. While the processor used for ACS and EACS algorithms is comparable to that used for GRASP-ELS and GVNS algorithms. This statement is based on the comparison of various computer systems solving standard linear equation problems presented in Dongarra (2014). The performance is evaluated on a benchmark problem of a dense system of linear equations given by a matrix of order 100 and 1000. The values for the processor used for GRASP-ELS and GVNS algorithms are 1571 and 3650 Mflop/s respectively; the values for the processor used for ACS and EACS algorithms are 1470 and 3654 Mflop/s respectively. The values for the processor used for IterLS algorithm are 2426 and 7519 Mflop/s respectively while the values for the processor used for VAN algorithm are 1317 and 2444 Mflop/s respectively. The values for the processor used for VNS which is an Intel Pentium 4 with 2.4 GHz are not available. However, the values for an Intel Pentium 4 with 2.5 GHz are available, this processor has achieved 1190 and 2355 Mflop/s respectively. Thus, we assume that the processor used for VNS algorithm is slower than that used for VAN algorithm. The values for the processor used for GA algorithm are not available. As only limited information was available on the processor used by GA, we cannot estimate its speed by considering only the clock-rate. A review of all processors i7 with 2.93 GHz shows that the Super pi ranges from 13.8 to 11.7 s. We assume that the processor used for DABC is comparable to that used for I3CH algorithm. We estimate the single-thread performance of each processor by supposing the performance of the machine of Gunawan et al. (2015) to be 1.

Table 3 summarizes the results obtained by the algorithms. It compares the average performance of HILS with that of the stat-of-the-art algorithms. Column Gap (%) reports the average percentual gap with the best known profit. Column CPU (s) reports the average computational time in seconds. The computational times of the algorithms are adjusted according to computers’ speed as presented in Table 2. The authors in Montemanni et al. (2011) did not report detailed results for their EACS algorithm, they have just reported average gaps with the former best known solutions. However, the authors in Gunawan et al. (2015) have recently reported the average gaps for the best algorithm among ACS and EACS called ACS* with the latest best known solutions. Thus, column ACS* of Table 3 presents the average result, over five runs, of ACS* algorithm.

We can see from Tables 1 and 3 that on Solomon instances with 100 customers, HILS algorithm performs, on average, better than GVNS and DABC on Class 1 (C100, R100 and RC100) both with regard to solution quality and computational time. On C100 and R100 test instances, HILS algorithm performs, on average, better than FSA, GA and IterLS using approximately the same computational effort. On these instances of Class 1, ILS, SSA and GRASP-ELS and I3CH are, on average, better than HILS at the cost of extra computational time. On the test instances of Class 2 (C200, R200 and RC200), HILS algorithm performs, on average, better than GA using more computational time. On C200 test instances, HILS performs, on average, better than GVNS both with regard to solution quality and computational time. On these instances of Class 2, most of state-of-the-art algorithms outperform HILS algorithm using more computational effort. On these test instances of Solomon with 100 customers, HILS achieved the optimal solution 19 times.

On the test instances of Cordeau, HILS algorithm is not as competitive as state-of-the-art algorithms on PR01-10 test instances; HILS algorithm achieved the worst average gap which is equal to 6.8 %. On PR11-20 test instances of Cordeau, HILS algorithm performs, on average, better than ACS* both with regard to solution quality and computational time and better than FSA using more computational effort. However, the rest of state-of-the-art algorithms perform, on average, better than HILS on these test instances of Cordeau. HILS algorithm is able to achieve 1 new best solution on PR17 test instance.

On test instances with 50 customers, HILS algorithm is as competitive as VAN algorithm both with regard to solution quality and computational time. VNS algorithm performs, on average, better than HILS and VAN algorithms at the expense of a reasonable amount of computational time. On these instances, HILS is able to achieve the optimal solution on 19 instances over 29.

**Table 1:** Results on the OPTW test instances

Instance-n	BKS	I3CH			ABC			VNS			ILS		
		Profit	Gap (%)	CPU (s)	Profit	Gap (%)	CPU (s)	Profit	Gap (%)	CPU (s)	Profit	Gap (%)	CPU (s)
C101-100	320	320	0.0	21.8	320	0.0	0.4	320	0.0	72.2	320	0.0	0.2
C102-100	360	360	0.0	28.1	360	0.0	0.6	360	0.0	102.2	360	0.0	0.3
C103-100	400	400	0.0	27.1	398	0.5	3.6	398	0.5	106.2	400	0.0	0.2
C104-100	420	420	0.0	27.1	420	0.0	15.2	418	0.5	124.3	420	0.0	0.4
C105-100	340	340	0.0	23.4	340	0.0	0.5	340	0.0	87.1	340	0.0	0.4
C106-100	340	340	0.0	23.6	340	0.0	0.5	340	0.0	86.7	340	0.0	0.5
C107-100	370	370	0.0	24.7	370	0.0	1.3	370	0.0	88.7	370	0.0	0.1
C108-100	370	370	0.0	24.8	370	0.0	0.9	370	0.0	99.3	370	0.0	0.5
C109-100	380	380	0.0	26.3	380	0.0	0.5	380	0.0	118.7	380	0.0	6.8
Avg			0.0	25.2		0.1	2.6		0.1	98.4		0.0	1.0
R101-100	198	198	0.0	20.4	190.0	4.2	0.3	198.0	0.0	49.9	198	0.0	0.1
R102-100	286	286	0.0	29.3	281.6	1.6	2.2	285.2	0.3	77.9	286	0.0	0.2
R103-100	293	293	0.0	28.8	292.0	0.3	1.1	293.0	0.0	90.2	293	0.0	1.4
R104-100	303	298	1.7	27.3	299.6	1.1	1.7	303.0	0.0	95.9	303	0.0	1.5
R105-100	247	247	0.0	26.0	242.6	1.8	9.1	247.0	0.0	85.4	247	0.0	0.7
R106-100	293	293	0.0	29.4	289.0	1.4	1.6	292.2	0.3	89.6	293	0.0	0.2
R107-100	299	297	0.7	27.8	299.0	0.0	7.1	299.0	0.0	105.5	299	0.0	0.5
R108-100	308	306	0.7	29.7	308.0	0.0	4.6	308	0.0	119.1	308	0.0	0.9
R109-100	277	277	0.0	31.1	277.0	0.0	1.6	277.0	0.0	77.4	277	0.0	0.2
R110-100	284	284	0.0	33.9	282.2	0.6	8.1	284.0	0.0	86.0	284	0.0	1.3
R111-100	297	295	0.7	27.7	294.0	1.0	1.7	297.0	0.0	95.4	297	0.0	10.9
R112-100	298	289	3.1	32.0	297.0	0.3	2.4	297.9	0.0	97.0	298	0.0	3.3
Avg			0.6	28.6		1.0	3.5		0.1	89.1		0.0	1.8
RC101-100	219	219	0.0	21.8	219	0.0	0.5	219	0.0	61.2	219	0.0	0.2
RC102-100	266	266	0.0	25.5	266	0.0	0.6	266	0.0	54.7	266	0.0	0.4
RC103-100	266	266	0.0	27.1	266	0.0	10.7	266	0.0	69.2	266	0.0	2.0
RC104-100	301	301	0.0	27.2	301	0.0	20.3	301	0.0	62.5	301	0.0	0.3
RC105-100	244	244	0.0	26.4	244	0.0	1.3	244	0.0	65.2	244	0.0	4.3
RC106-100	252	250	0.8	25.0	251.4	0.2	8.1	252	0.0	67.4	252	0.0	0.3
RC107-100	277	274	1.1	26.3	276.2	0.3	10.4	277	0.0	72.4	277	0.0	0.3
RC108-100	298	264	12.9	25.1	298	0.0	11.3	298	0.0	71.5	298	0.0	0.1
Avg			1.9	25.6		0.1	7.9		0.0	65.5		0.0	1.0
PR01-48	308	305	1.0	20.8	307	0.3	5.8	308	0.0	77.9	–	–	–
PR02-96	404	394	2.5	47.9	391.2	3.2	4.9	403.9	0.0	244.6	–	–	–
PR03-144	394	394	0.0	72.9	394	0.0	12.6	390.5	0.9	387.2	–	–	–
PR04-192	489	489	0.0	109.3	486	0.6	67.4	488.1	0.2	541.6	–	–	–
PR05-240	595	594	0.2	185.4	586.8	1.4	91.4	586.1	1.5	1455.2	–	–	–
PR06-288	591	590	0.2	189.9	573.6	2.9	247.4	588.2	0.5	1633.8	–	–	–
PR07-72	298	298	0.0	26.5	298	0.0	4.3	297.5	0.2	131.0	–	–	–
PR08-144	463	454	1.9	77.4	462	0.2	15.0	452.2	2.3	514.1	–	–	–
PR09-216	493	490	0.6	137.8	481.8	2.3	126.3	481.5	2.3	920.8	–	–	–
PR10-288	594	568	4.4	222.2	570.4	4.0	210.3	575.5	3.1	1534.1	–	–	–
Avg			1.1	109.0		1.5	78.5		1.1	744.0			
C201-100	870	870	0.0	70.1	870	0.0	11.2	870	0.0	507.8	870	0.0	36.7
C202-100	930	930	0.0	87.6	930	0.0	15.3	928	0.2	454.8	930	0.0	59.0
C203-100	960	960	0.0	92.3	958	0.2	14.8	960	0.0	614.3	960	0.0	137.2
C204-100	980	970	1.0	117.4	966	1.4	25.7	972	0.8	484	974	0.6	217.6
C205-100	910	900	1.1	70.7	900	1.1	1.7	908	0.2	645.4	908	0.2	56.2
C206-100	930	920	1.1	75.7	928	0.2	6.2	927	0.3	616.5	927	0.3	111.5
C207-100	930	930	0.0	77.4	918	1.3	2.3	930	0.0	599.5	930	0.0	68.1
C208-100	950	950	0.0	84.0	950	0.0	11.8	949	0.1	558.9	950	0.0	33.3
Avg			0.4	84.4		0.5	11.1		0.2	560.2		0.1	90.0
R201-100	797	789	1.0	101.8	787	1.3	20.3	796.7	0.0	1021.7	788.7	1.0	133.7
R202-100	930	930	0.0	175.6	895.8	3.7	40.6	905.6	2.6	1057.5	910.3	2.1	165.6
R203-100	1028	1020	0.8	221.4	1009	1.8	25.6	1007.9	2.0	1139.9	1011.3	1.6	213.5
R204-100	1093	1073	1.8	236.9	1070.4	2.1	34.0	1076.3	1.5	1253.5	1082.8	0.9	171.0
R205-100	953	946	0.7	129.3	953	0.0	32.9	952.6	0.0	745.1	948.4	0.5	169.9
R206-100	1032	1021	1.1	169.3	1018	1.4	21.3	1014	1.7	1168.1	1012.4	1.9	126.5
R207-100	1077	1050	2.5	192.8	1060.8	1.5	20.9	1061.5	1.4	1065.3	1059.5	1.6	174.0
R208-100	1117	1092	2.2	230.0	1084.6	2.9	26.7	1101.5	1.4	1160.8	1107.6	0.8	165.6
R209-100	959	948	1.1	136.5	934.6	2.5	26.3	947.8	1.2	925.9	949.7	1.0	145.8
R210-100	991	982	0.9	176.9	965.4	2.6	29.3	975.9	1.5	1065.6	970.8	2.0	171.8
R211-100	1051	1013	3.6	167.4	1019	3.0	15.0	1024.2	2.5	1120.7	1040.4	1.0	145.7
Avg			1.4	176.2		2.1	26.6		1.4	1065.8		1.3	162.1
RC201-100	795	795	0.0	80.9	784	1.4	24.9	795	0.0	640.8	795	0.0	63.5
RC202-100	938	924	1.5	129.3	926.8	1.2	27.3	925.6	1.3	951.5	929	1.0	156.2
RC203-100	1003	966	3.7	134.3	962.4	4.0	24.8	988.2	1.5	938.4	989.8	1.3	111.5
RC204-100	1140	1093	4.1	167.5	1109.2	2.7	23.4	1120.8	1.7	970.3	1131.3	0.8	165.0
RC205-100	859	847	1.4	99.2	852.3	0.8	27.2	845.9	1.5	726.6	854.7	0.5	100.5
RC206-100	899	863	4.0	98.4	890.2	1.0	24.9	878.4	2.3	838.7	894.1	0.5	152.0
RC207-100	983	957	2.6	122.0	977.0	0.6	15.8	960.8	2.3	893.5	952.1	3.1	129.9
RC208-100	1057	1003	5.1	123.0	1032.8	2.3	26.3	1043.5	1.3	995.5	1040.7	1.5	85.6
Avg			2.8	119.3		1.8	24.3		1.5	869.4		1.1	120.5
PR11-48	353	353	0.0	30.8	350.6	0.7	1.7	328	7.1	131.4	–	–	–
PR12-96	442	433	2.0	59.8	432	2.3	23.5	442	0.0	309.6	–	–	–
PR13-144	467	466	0.2	89.5	458.1	1.9	8.9	453.7	2.8	514.1	–	–	–
PR14-192	567	521	8.1	144.4	560.1	1.2	76.6	550.1	3.0	1163.8	–	–	–
PR15-240	708	707	0.1	248.2	666.4	5.9	274.6	663.2	6.3	1900.9	–	–	–
PR16-288	674	619	8.2	228.6	613.4	9.0	205.1	637	5.5	1854.1	–	–	–
PR17-72	362	360	0.6	34.7	359	0.8	6.6	358.3	1.0	140.5	–	–	–
PR18-144	539	497	7.8	99.0	535	0.7	22.1	519.4	3.6	760.9	–	–	–
PR19-216	562	538	4.3	164.6	541	3.7	70.7	551.6	1.9	1242.2	–	–	–
PR20-288	667	588	11.8	202.7	604.8	9.3	346.9	647.8	2.9	2441.9	–	–	–
Avg			4.3	130.2		3.6	103.7		3.4	1045.9			

Instance-n	BKS	ACS			GVNS			GRASP-ELS			SSA		
		Profit	Gap (%)	CPU (s)	Profit	Gap (%)	CPU (s)	Profit	Gap (%)	CPU (s)	Profit	Gap (%)	CPU (s)
C101-100	320	320	0.0	0.5	320	0.0	0.2	320	0.0	1.2	320	0.0	20.4
C102-100	360	360	0.0	0.7	360	0.0	67.4	360	0.0	16.5	360	0.0	20.7
C103-100	400	400	0.0	16.9	396	1.0	382.4	400	0.0	38.5	400	0.0	24.2
C104-100	420	420	0.0	33.5	410	2.4	1005.9	420	0.0	105.2	420	0.0	21.9
C105-100	340	340	0.0	0.9	340	0.0	3.3	340	0.0	2.3	340	0.0	20.6
C106-100	340	340	0.0	1.0	340	0.0	6.5	340	0.0	3.8	340	0.0	20.3
C107-100	370	370	0.0	2.1	358	3.4	0.7	370	0.0	3.7	370	0.0	20.5
C108-100	370	370	0.0	0.8	354	4.5	1.1	370	0.0	6.6	370	0.0	20.5
C109-100	380	380	0.0	0.8	380	0.0	30.6	380	0.0	25.5	380	0.0	20.5
Avg			0.0	6.4		1.3	166.5		0.0	22.6		0.0	21.1
R101-100	198	198	0.0	0.1	197	0.5	0.2	198	0.0	0.9	198	0.0	19.7
R102-100	286	286	0.0	11.1	274.8	4.1	13.4	286	0.0	2.3	286	0.0	21
R103-100	293	292.6	0.1	640.6	286	2.4	33.8	290.4	0.9	4.8	293	0.0	20.3
R104-100	303	303	0.0	164.0	298.6	1.5	72.7	302.8	0.1	5.1	303	0.0	23.2
R105-100	247	247	0.0	3.0	230.6	7.1	0.3	247	0.0	1.2	247	0.0	20.3
R106-100	293	293	0.0	86.3	280.4	4.5	19.1	293	0.0	3.3	293	0.0	22.1
R107-100	299	294.6	1.5	922.6	287.2	4.1	50.3	296.4	0.9	4.8	297	0.7	21.5
R108-100	308	306	0.7	696.1	301.4	2.2	50.1	306.8	0.4	5.0	306	0.7	40.6
R109-100	277	277	0.0	28.0	276.4	0.2	10.4	277	0.0	2.7	277	0.0	20.4
R110-100	284	283.2	0.3	617.6	279.2	1.7	16.8	283.6	0.1	2.8	284	0.0	20.8
R111-100	297	296.6	0.1	484.4	290.6	2.2	54.2	297	0.0	4.6	297	0.0	27.9
R112-100	298	297.4	0.2	947.1	289.6	2.9	31.9	297.2	0.3	4.6	298	0.0	22.3
Avg			0.2	383.4		2.8	29.4		0.2	3.5		0.1	23.3
RC101-100	219	219	0.0	0.2	219	0.0	2.1	219	0.0	0.6	219	0.0	19.8
RC102-100	266	266	0.0	30.9	246	8.1	5.8	259	2.7	2.1	266	0.0	20.2
RC103-100	266	266	0.0	57.2	253.2	5.1	23.3	265.2	0.3	2.7	266	0.0	20.7
RC104-100	301	301	0.0	29.4	301	0.0	16.2	300.2	0.3	3.1	301	0.0	27.5
RC105-100	244	244	0.0	9.7	227	7.5	2.7	244	0.0	1.4	244	0.0	20.3
RC106-100	252	252	0.0	308.6	252	0.0	2.2	252	0.0	1.2	252	0.0	21
RC107-100	277	277	0.0	502.1	261.2	6.0	15.7	277	0.0	1.9	277	0.0	22
RC108-100	298	298	0.0	207.5	288.8	3.2	10.4	298	0.0	2.9	298	0.0	26
Avg			0.0	143.2		3.7	9.8		0.4	2.0		0.0	22.2
PR01-48	308	308	0.0	256.2	307.2	0.3	1.2	308	0.0	1.2	305	1.0	8.3
PR02-96	404	403.8	0.0	1147.8	403.6	0.1	3.7	402.6	0.3	3.0	404	0.0	29.1
PR03-144	394	394	0.0	2024.7	388	1.5	4.1	394	0.0	3.1	394	0.0	59.9
PR04-192	489	482.6	1.3	1404.7	475.4	2.8	14.7	474.6	2.9	5.3	489	0.0	106.7
PR05-240	595	576.8	3.1	2075.7	578	2.9	20.5	581	2.4	9.1	589	1.0	281.7
PR06-288	591	564.6	4.5	2199.8	584.2	1.2	29.0	583.4	1.3	8.6	575	2.7	253.4
PR07-72	298	298	0.0	20.1	297	0.3	1.7	294.4	1.2	1.6	298	0.0	15.0
PR08-144	463	462.6	0.1	2476.0	463	0.0	3.8	462.8	0.0	3.3	462	0.2	76.0
PR09-216	493	481.8	2.3	2318.2	482	2.2	12.3	477.8	3.1	7.0	482	2.2	102.3
PR10-288	594	588.4	0.9	2343.5	564.4	5.0	32.7	574.2	3.3	8.1	578	2.7	189.7
Avg			1.2	1626.7		1.6	12.4		1.5	5.0		1.0	112.2
C201-100	870	870	0.0	189.8	850	2.3	0.1	870	0.0	2.5	870	0.0	28.3
C202-100	930	930	0.0	319.0	916	1.5	78.7	910	2.2	20.4	930	0.0	33.5
C203-100	960	960	0.0	361.0	956	0.4	329	960	0.0	45.2	960	0.0	59.6
C204-100	980	970	1.0	1617.5	966	1.4	974	970	1.0	130.3	970	1.0	42.3
C205-100	910	906	0.4	28.2	898	1.3	12.5	906	0.4	10.6	910	0.0	46.9
C206-100	930	920	1.1	87.9	922	0.9	34.4	926	0.4	13.4	930	0.0	29
C207-100	930	920	1.1	48.5	928	0.2	36.3	930	0.0	17.3	930	0.0	29.1
C208-100	950	940.2	1.0	89.0	942	0.8	74.2	942	0.8	17.7	950	0.0	31.2
Avg			0.6	342.6		1.1	192.4		0.6	32.2		0.1	37.5
R201-100	797	795.8	0.2	2339.9	775.6	2.7	6.7	788.2	1.1	5.3	794	0.4	51.1
R202-100	930	899.6	3.3	1724.4	881.4	5.2	13.4	909.8	2.2	9.6	914	1.7	46.4
R203-100	1028	989.9	3.7	2641.7	992.2	3.5	34.7	1001.4	2.6	12.6	997	3.0	44.2
R204-100	1093	1046.4	4.3	1549.1	1073.8	1.8	77.6	1071.8	1.9	15.2	1058	3.2	39.7
R205-100	953	939.2	1.4	1125.4	905.8	5.0	15.3	927.2	2.7	7.8	946	0.7	37.8
R206-100	1032	983.0	4.7	1671.4	966.6	6.3	34.9	1023.6	0.8	10.7	1020	1.2	40.9
R207-100	1077	1026.6	4.7	1090.3	1022	5.1	46.6	1049.2	2.6	13.5	1069	0.7	52.5
R208-100	1117	1057.2	5.4	1260.9	1083.6	3.0	52.9	1100.2	1.5	15.9	1079	3.4	35.8
R209-100	959	923.4	3.7	1453.2	926	3.4	37.3	937.4	2.3	10.3	945	1.5	61.6
R210-100	991	956.4	3.5	979.1	961.4	3.0	30.1	969	2.2	10.8	973	1.8	53.6
R211-100	1051	1006.8	4.2	1288.1	1025.6	2.4	22.5	1028.2	2.2	11.3	1041	1.0	40.5
Avg			3.5	1556.7		3.8	33.8		2.0	11.2		1.7	45.8
RC201-100	795	795	0.0	640.8	784	1.4	3.7	786.4	1.1	4.2	795	0.0	44.4
RC202-100	938	931.6	0.7	951.5	890.6	5.1	12.1	922.8	1.6	8.9	930	0.9	46.3
RC203-100	1003	976.6	2.6	938.4	954.4	4.8	22.5	978.2	2.5	9.6	967	3.6	32.4
RC204-100	1140	1086.2	4.7	970.3	1101.8	3.4	31.9	1096.0	3.9	11.9	1140	0.0	46.5
RC205-100	859	848.4	1.2	726.6	843.8	1.8	7	844.6	1.7	6.8	854	0.6	52.6
RC206-100	899	884.0	1.7	838.7	866.6	3.6	11.6	886.8	1.4	6.1	885	1.6	60.2
RC207-100	983	960.8	2.3	893.5	911.4	7.3	14.7	965.4	1.8	7.5	977	0.6	68.4
RC208-100	1057	1013.2	4.1	995.5	1000.2	5.4	24.6	1007.0	4.7	10.7	1041	1.5	51.2
Avg			2.2	869.4		4.1	16.0		2.3	8.2		1.1	50.3
PR11-48	353	327.8	7.1	1743.7	329	6.8	1.9	329.2	6.7	2.0	351.0	0.6	10.3
PR12-96	442	436.4	1.3	2017.6	435	1.6	6.5	442.0	0.0	4.0	430.0	2.7	26.3
PR13-144	467	441.0	5.6	2312.7	452.4	3.1	16.2	456.6	2.2	4.9	452.0	3.2	49.0
PR14-192	567	494.0	12.9	23.1	540.6	4.7	32.4	541.2	4.6	8.8	540.0	4.8	134.3
PR15-240	708	524.8	25.9	18.2	656.6	7.3	29.4	665.0	6.1	13.1	666.0	5.9	118.5
PR16-288	674	517.8	23.2	25.7	643.4	4.5	60.9	652.6	3.2	16.2	616.0	8.6	558.0
PR17-72	362	358.0	1.1	1330.9	354.6	2.0	5.4	356.2	1.6	2.4	362.0	0.0	37.6
PR18-144	539	488.8	9.3	1350.0	530.8	1.5	10.4	517.6	4.0	5.0	539.0	0.0	61.8
PR19-216	562	475.0	15.5	30.0	507.8	9.6	22.1	537.8	4.3	8.7	531.0	5.5	152.9
PR20-288	667	552.4	17.2	24.6	655.0	1.8	57.0	656.6	1.6	13.9	626.0	6.1	475.3
Avg			11.9	887.7		4.3	24.2		3.4	7.9		3.7	162.4

Instance-n	BKS	IterLS			FSA			DABC			HILS		
		Profit	Gap (%)	CPU (s)	Profit	Gap (%)	CPU (s)	Profit	Gap (%)	CPU (s)	Profit	Gap (%)	CPU (s)
C101-100	320	320	0.0	0.4	320	0.0	0.4	312	2.5	0.4	320	0.0	0.4
C102-100	360	360	0.0	0.3	360	0.0	0.3	360	0.0	98.6	360	0.0	0.4
C103-100	400	390	2.6	0.5	390	2.5	0.5	390	2.5	1.0	390	2.5	0.4
C104-100	420	400	5.0	0.3	410	2.4	0.3	420	0.0	10.4	420	0.0	0.7
C105-100	340	340	0.0	0.3	330	2.9	0.3	322	5.3	1.2	340	0.0	0.4
C106-100	340	340	0.0	0.3	340	0.0	0.3	340	0.0	2.0	340	0.0	0.4
C107-100	370	360	2.8	0.3	370	0.0	0.3	350	5.4	1.2	370	0.0	0.4
C108-100	370	370	0.0	0.3	370	0.0	0.3	370	0.0	18.8	370	0.0	0.4
C109-100	380	380	0.0	0.3	380	0.0	0.3	380	0.0	22.0	380	0.0	0.4
Avg			1.2	0.3		0.9	0.3		1.7	17.3		0.3	0.4
R101-100	198	182	8.8	0.1	198	0.0	0.1	182.2	8.0	0.6	198	0.0	0.2
R102-100	286	286	0.0	0.2	282	1.4	0.2	286	0.0	5.4	286	0.0	0.6
R103-100	293	286	2.4	0.2	293	0.0	0.2	293	0.0	49.4	287	2.0	0.7
R104-100	303	297	2.0	0.2	294	3.0	0.2	303	0.0	15	299	1.3	0.7
R105-100	247	247	0.0	0.1	247	0.0	0.1	243.4	1.5	62.2	247	0.0	0.3
R106-100	293	293	0.0	0.2	270	7.8	0.2	293	0.0	0.8	293	0.0	0.6
R107-100	299	288	3.8	0.2	277	7.4	0.2	297	0.7	1.4	299	0.0	0.7
R108-100	308	297	3.7	0.2	294	4.5	0.2	306	0.6	31	308	0.0	0.7
R109-100	277	276	0.4	0.2	264	4.7	0.2	233.4	15.7	0.6	276	0.4	0.4
R110-100	284	281	1.1	0.3	282	0.7	0.3	281.2	1.0	1.0	281	1.1	0.4
R111-100	297	295	0.7	0.2	286	3.7	0.2	295	0.7	85.4	294	1.0	0.7
R112-100	298	295	1.0	0.2	284	4.7	0.2	290.8	2.4	50.8	295	1.0	0.7
Avg			2.0	0.2		3.2	0.2		2.6	25.3		0.6	0.6
RC101-100	219	219	0.0	0.2	216	1.4	0.2	206	5.9	2.2	219	0.0	0.2
RC102-100	266	259	2.7	0.2	249	6.4	0.2	259	2.6	1.0	259	2.6	0.3
RC103-100	266	265	0.4	0.3	265	0.4	0.3	255.8	3.8	6.4	248	6.8	0.3
RC104-100	301	297	1.3	0.3	263	12.6	0.3	288.4	4.2	86.8	275	8.6	0.3
RC105-100	244	221	10.4	0.2	219	10.2	0.2	228	6.6	4.4	244	0.0	0.3
RC106-100	252	239	5.4	0.2	240	4.8	0.2	225	10.7	0.6	244	3.2	0.3
RC107-100	277	274	1.1	0.2	244	11.9	0.2	262.4	5.3	45	276	0.4	0.3
RC108-100	298	288	3.5	0.2	263	11.7	0.2	284	4.7	82.6	278	6.7	0.3
Avg			3.1	0.2		7.4	0.2		5.5	28.6		3.5	0.3
PR01-48	308	304	1.3	0.5	304	1.3	0.5	–	–	–	308	0.0	1.1
PR02-96	404	385	4.7	0.6	392	3.0	0.6	–	–	–	397	1.7	1.2
PR03-144	394	384	2.5	1.0	381	3.3	1.0	–	–	–	378	4.1	1.4
PR04-192	489	447	8.6	1.9	470	3.9	1.9	–	–	–	452	7.6	2.1
PR05-240	595	576	3.2	4.6	527	11.4	4.6	–	–	–	523	12.4	5.5
PR06-288	591	538	9.0	2.5	557	5.8	2.5	–	–	–	496	16.1	5.8
PR07-72	298	291	2.3	0.4	289	3.0	0.4	–	–	–	285	10.7	0.4
PR08-144	463	463	0.0	1.0	438	5.4	1.0	–	–	–	455	1.7	2.1
PR09-216	493	461	6.5	1.4	461	6.5	1.4	–	–	–	447	9.3	2.2
PR10-288	594	539	9.3	3.6	539	9.3	3.6	–	–	–	526	11.4	4.9
Avg			4.7	1.8		5.3	1.8					6.8	2.7
C201-100	870	840	3.4	1.1	870	0.0	1.1	840	3.4	23.8	870	0.0	3.9
C202-100	930	910	2.2	2.8	930	0.0	2.8	930	0.0	127.4	910	2.2	5.7
C203-100	960	940	2.1	1.7	940	2.1	1.7	960	0.0	153.4	960	0.0	6.2
C204-100	980	950	3.1	1.6	950	3.1	1.6	970	1.0	117.6	970	1.0	9.2
C205-100	910	900	1.1	1.2	900	1.1	1.2	906	0.4	23.6	900	1.1	5.3
C206-100	930	910	2.2	1.6	920	1.1	1.6	928	0.2	142	920	1.1	5.9
C207-100	930	910	2.2	2.1	930	0.0	2.1	930	0.0	142.4	920	1.1	6.1
C208-100	950	930	2.1	1.6	940	1.1	1.6	950	0.0	132.2	950	0.0	6.1
Avg			2.3	1.7		1.1	1.7		0.6	107.8		0.8	6.0
R201-100	797	788	1.1	1.2	772	3.1	1.2	784	1.6	49.4	781	2.0	6.1
R202-100	930	880	5.4	1.4	878	5.6	1.4	912.8	1.8	105.4	890	4.3	14.3
R203-100	1028	980	4.7	1.6	988	3.9	1.6	1019.6	0.8	218.6	970	5.6	22.4
R204-100	1093	1073	1.8	1.7	1059	3.1	1.7	1089.4	0.3	77.4	1037	5.1	29.6
R205-100	953	931	2.3	1.4	936	1.8	1.4	951.4	0.2	129.0	894	6.2	11.2
R206-100	1032	996	3.5	1.5	950	7.9	1.5	1024.8	0.7	187.8	994	3.7	18.4
R207-100	1077	1038	3.6	2.0	1033	4.1	2.0	1070.6	0.6	142.2	1015	5.8	23.5
R208-100	1117	1069	4.3	1.6	1066	4.6	1.6	1114	0.3	130.2	1061	5.0	33.7
R209-100	959	926	3.4	2.4	914	4.7	2.4	948.4	1.1	164.4	892	7.0	14.3
R210-100	991	958	3.3	1.9	975	1.6	1.9	972	1.9	170.4	929	6.3	16.3
R211-100	1051	1023	2.7	1.6	1023	2.7	1.6	1037.2	1.3	116.8	996	5.2	21.5
Avg			3.3	1.7		3.9	1.7		1.0	135.6		5.1	19.2
RC201-100	795	780	1.9	1.0	781	1.8	1.0	792	0.4	65.6	771	3.0	1.8
RC202-100	938	882	6.0	1.3	866	7.7	1.3	924.4	1.4	135.2	905	3.5	6.2
RC203-100	1003	960	4.3	2.7	956	4.7	2.7	983.4	2.0	138.0	947	5.6	10.3
RC204-100	1140	1117	2.0	2.3	1061	6.9	2.3	1130.8	0.8	168.8	1068	6.3	19.4
RC205-100	859	840	2.2	1.0	800	6.9	1.0	841.2	2.1	119.2	820	4.5	6.0
RC206-100	899	860	4.3	1.1	866	3.7	1.1	884.8	1.6	81.4	851	5.3	6.1
RC207-100	983	926	5.8	1.3	899	8.5	1.3	937.6	4.6	117.2	923	6.1	9.2
RC208-100	1057	1037	1.9	2.3	1015	4.0	2.3	1041.0	1.5	34.0	1008	4.6	13.3
Avg			3.6	1.6		5.5	1.6		1.8	107.4		4.9	9.0
PR11-48	353	330	6.5	0.3	339	4.0	0.3	–	–	–	351	0.6	0.7
PR12-96	442	431	2.5	0.9	432	2.3	0.9	–	–	–	427	3.4	2.1
PR13-144	467	450	3.6	1.9	424	9.2	1.9	–	–	–	436	6.6	2.7
PR14-192	567	482	15.0	1.1	499	12.0	1.1	–	–	–	479	15.5	6.0
PR15-240	708	638	9.9	5.3	613	13.4	5.3	–	–	–	616	13.0	16.4
PR16-288	674	559	17.1	4.1	562	16.6	4.1	–	–	–	582	13.6	13.3
PR17-72	362	346	4.4	0.2	335	7.5	0.2	–	–	–	*363*	−0.3	1.0
PR18-144	539	479	11.1	0.8	477	11.5	0.8	–	–	–	459	14.8	4.1
PR19-216	562	499	11.2	2.7	501	10.9	2.7	–	–	–	479	14.8	6.2
PR20-288	667	570	14.5	2.5	568	14.8	2.5	–	–	–	588	11.8	10.2
Avg			9.6	2.0		10.2	2.0					9.4	6.3

Instance-n	BKS	GA											
		Profit	Gap (%)	CPU (s)									
C101-100	320	317	0.9	0.3									
C102-100	360	360	0.0	0.3									
C103-100	400	389	2.8	0.4									
C104-100	420	403	4.0	0.4									
C105-100	340	340	0.0	0.3									
C106-100	340	340	0.0	0.3									
C107-100	370	362	2.2	0.3									
C108-100	370	370	0.0	0.3									
C109-100	380	380	0.0	0.3									
Avg			1.1	0.3									
R101-100	198	189	4.5	0.2									
R102-100	286	286	0.0	0.3									
R103-100	293	290	1.0	0.3									
R104-100	303	297	2.0	0.3									
R105-100	247	244	1.2	0.2									
R106-100	293	292	0.3	0.3									
R107-100	299	292	2.3	0.4									
R108-100	308	300	2.6	0.3									
R109-100	277	270	2.5	0.3									
R110-100	284	277	2.5	0.3									
R111-100	297	293	1.3	0.4									
R112-100	298	293	1.7	0.4									
Avg			1.8	0.3									
RC101-100	219	216	1.4	0.2									
RC102-100	266	261	1.9	0.3									
RC103-100	266	262	1.5	0.3									
RC104-100	301	294	2.3	0.4									
RC105-100	244	236	3.3	0.3									
RC106-100	252	245	2.8	0.3									
RC107-100	277	269	2.9	0.3									
RC108-100	298	289	3.0	0.3									
Avg			2.4	0.3									
C201-100	870	848	2.5	0.8									
C202-100	930	901	3.1	1.0									
C203-100	960	931	3.0	1.1									
C204-100	980	952	2.9	1.5									
C205-100	910	893	1.9	1.0									
C206-100	930	905	2.7	1.0									
C207-100	930	910	2.2	1.0									
C208-100	950	931	2.0	1.0									
Avg			2.5	1.0									
R201-100	797	760	4.6	1.2									
R202-100	930	867	6.8	1.8									
R203-100	1028	954	7.2	2.2									
R204-100	1093	1018	6.9	2.5									
R205-100	953	876	8.1	1.6									
R206-100	1032	954	7.6	2.0									
R207-100	1077	986	8.4	2.1									
R208-100	1117	1042	6.7	2.4									
R209-100	959	897	6.5	1.7									
R210-100	991	915	7.7	1.8									
R211-100	1051	967	8.0	2.4									
Avg			7.1	2.0									
RC201-100	795	768	3.4	1.3									
RC202-100	938	866	7.7	1.3									
RC203-100	1003	866	13.7	1.3									
RC204-100	1140	1044	8.4	2.0									
RC205-100	859	808	5.9	1.3									
RC206-100	899	850	5.5	1.3									
RC207-100	983	896	8.9	1.7									
RC208-100	1057	976	7.7	1.4									
Avg			7.6	1.5									

Instance-n	Opt		VAN		VNS				HILS				
	Profit	CPU (s)	Profit	CPU (s)	Worst	Best	Avg	CPU (s)	Best	Worst	Avg	CPU (s)	
C101-50	270	0.0	270	0.3	270	270	270	44.5	270	270	270	0.2	
C102-50	300	1.1	300	0.3	300	300	300	65.4	290	290	290	0.2	
C103-50	320	23.0	320	0.3	320	320	320	69.0	310	310	310	0.4	
C104-50	340	81.4	340	0.3	330	340	340	80.7	340	340	340	0.4	
C105-50	300	0.0	300	0.3	300	300	300	45.2	300	300	300	0.2	
C106-50	280	0.0	280	0.2	280	280	280	39.7	280	280	280	0.2	
C107-50	310	0.0	310	0.2	310	310	310	53.8	310	310	310	0.2	
C108-50	320	0.2	320	0.3	320	320	320	42.7	320	320	320	0.2	
C109-50	340	0.9	340	0.2	340	340	340	41.7	340	340	340	0.3	
Avg CPU (s)		11.8		0.3				53.6				0.3	
Adjusted CPU (s)				0.1				<11.8				0.1	
R101-50	126	0.0	126	0.1	126	126	126	13.6	126	126	126	0.1	
R102-50	198	0.7	195	0.2	198	198	198	24.5	195	195	195	0.2	
R103-50	214	30.0	210	0.2	214	214	214	26.5	214	214	214	0.2	
R104-50	227	152.7	227	0.3	227	227	227	29.3	227	227	227	0.3	
R105-50	159	0.0	159	0.1	159	159	159	16.1	159	159	159	0.2	
R106-50	208	0.6	203	0.2	208	208	208	24.8	203	203	203	0.1	
R107-50	220	9.8	220	0.3	219	220	219.9	32.5	219	219	219	0.2	
R108-50	227	410.6	223	0.2	227	227	227	28.2	227	227	227	0.2	
R109-50	192	0.2	192	0.2	192	192	192	20.1	192	192	192	0.3	
R110-50	208	1.6	208	0.2	208	208	208	23.4	208	208	208	0.2	
R111-50	223	1.8	223	0.2	223	223	223	25.2	223	223	223	0.3	
R112-50	226	3.1	226	0.2	226	226	226	26.0	226	226	226	0.3	
Avg CPU (s)		50.9		0.2				24.2				0.2	
Adjusted CPU (s)				0.0				<5.3				0.0	
RC101-50	180	0.0	180	0.1	180	180	180	30.8	180	180	180	0.1	
RC102-50	230	0.7	230	0.2	230	230	230	32.8	230	230	230	0.1	
RC103-50	240	2.2	240	0.2	240	240	240	32.5	230	230	230	0.1	
RC104-50	270	6.1	270	0.2	260	270	266	38.3	250	250	250	0.2	
RC105-50	210	0.7	200	0.2	210	210	210	22.2	210	210	210	0.1	
RC106-50	210	0.8	200	0.2	210	210	210	33.9	200	200	200	0.1	
RC107-50	240	3.4	230	0.1	240	240	240	31.1	220	220	220	0.2	
RC108-50	250	9.3	240	0.2	250	250	250	33.8	240	240	240	0.2	
Avg CPU (s)		2.9		0.2				31.9				0.1	
Adjusted CPU (s)				0.0				<7.0				0.0	

The new best solutions obtained by HILS algorithm are presented as italics numbers

**Table 2:** Estimate of single-thread performance

Algorithm	Experimental environment	Super Pi	Estimate of single-thread performance
ACS	Dual AMD Opteron 250 2.4 GHz CPU, 4 GB RAM	Unknown	0.22
EACS	Dual AMD Opteron 250 2.4 GHz CPU, 4 GB RAM	Unknown	0.22
IterLS	Intel core 2 2.5 GHz CPU, 3.45 GB RAM	18.6	0.53
VNS	2.4 GHz CPU, 4 GB RAM	Unknown	<0.22
GRASP-ELS	Intel Pentium 4 processor, 3.00 GHz, 1 GB RAM	44.3	0.22
SSA	Intel Core 2 CPU, 2.5 GHz	18.6	0.53
FSA	Intel Core 2 CPU, 2.5 GHz	18.6	0.53
GVNS	Intel Pentium (R) IV, 3 GHz CPU	44.3	0.22
I3CH	Intel Xeon E5430 CPU clocked at 2.66 GHz, 8 GB RAM	14.7	0.67
HILS	Intel(R) Pentium(R) CPU B950, 2.1 GHz, 4 GB RAM	23	0.43
ABC	AMD Athlon X2 250 3.00 GHz	32.1	0.31
ILS	Intel Core i7-4770 with 3.4 GHz, 16 GB RAM	9.8	1
GA	Intel Core i7, 1.73 GHz CPU (turbo boost to 2.93 GHz)	Unknown	≤0.70
DABC	Intel Core 2 Quad processor with 2.66 GHz CPU	Unknown	≤0.67
VAN	Intel Pentium 4 with 2.8 GHz, 1 GB RAM	Unknown	<0.22

**Table 3:** Overall comparison on OPTW test instances

Set	I3CH		ABC		VNS		ILS		ACS		GVNS	
	Gap (%)	CPU (s)	Gap (%)	CPU (s)	Gap (%)	CPU (s)	Gap (%)	CPU (s)	Gap (%)	CPU (s)	Gap (%)	CPU (s)
C100-100	0.0	16.9	0.1	0.8	0.1	<21.6	0.0	1.0	0.0	1.4	1.3	36.6
R100-100	0.6	19.2	1.0	1.1	0.1	<19.6	0.0	1.8	0.2	84.3	2.8	6.5
RC100-100	1.9	17.2	0.1	2.4	0.0	<14.4	0.0	1.0	0.0	31.5	3.7	2.2
PR01-10	1.1	73.0	1.5	24.3	1.1	<163.7	0.7	50.4	1.2	357.9	1.6	2.7
C200-100	0.4	56.5	0.5	3.4	0.2	<123.2	0.1	90.0	0.6	75.4	1.1	42.3
R200-100	1.4	118.1	2.1	8.2	1.4	<234.5	1.3	162.1	3.5	342.5	3.8	7.4
RC200-100	2.8	79.9	1.8	7.5	1.5	<191.3	1.1	120.5	2.2	191.3	4.1	3.5
PR11-20	4.3	87.2	3.6	32.4	3.4	<230.1	2.1	97.9	11.9	195.3	4.3	5.3

Set	GRASP-ELS		SSA		IterLS		FSA		DABC		HILS	
	Gap (%)	CPU (s)	Gap (%)	CPU (s)	Gap (%)	CPU (s)	Gap (%)	CPU (s)	Gap (%)	CPU (s)	Gap (%)	CPU (s)
C100-100	0.0	5.0	0.0	11.2	1.2	0.2	0.9	0.3	1.7	≤11.6	0.3	0.2
R100-100	0.2	0.8	0.1	12.3	2.0	0.2	3.2	0.1	2.6	≤17.0	0.6	0.3
RC100-100	0.4	0.4	0.0	11.8	3.1	0.2	7.4	0.1	5.5	≤19.2	3.5	0.1
PR01-10	1.5	1.1	1.0	59.5	4.7	1.0	5.3	1.0	–	–	6.8	1.2
C200-100	0.6	7.1	0.1	19.9	2.3	0.9	1.1	0.9	0.6	≤72.2	0.8	2.6
R200-100	2.0	2.5	1.7	24.3	3.3	0.9	3.9	0.9	1.0	≤90.9	5.1	8.3
RC200-100	2.3	1.8	1.1	26.7	3.6	0.8	5.5	0.8	1.8	≤72.0	4.9	3.9
PR11-20	3.4	1.7	3.7	86.1	9.6	1.1	10.2	1.1	–	–	9.4	2.7

Set	GA		ACS*									
	Gap (%)	CPU (s)	Gap (%)	CPU (s)								
C100-100	1.1	≤0.2	0.0	1.4								
R100-100	1.8	≤0.2	0.2	84.8								
RC100-100	2.4	≤0.2	0.0	31.7								
PR01-10	–	–	1.2	359.8								
C200-100	2.5	≤0.7	0.6	75.8								
R200-100	7.1	≤1.4	3.2	344.4								
RC200-100	7.6	≤1.1	2.0	341.7								
PR11-20	–	–	11.9	196.4

**Table 4:** Results on the OPSTW test instances

Instance	$(P_{max}, W_{max})$	HILS						
		Best	Worst	Average	CPU (s)	$N_R$	%HTW	Routed customers
PR01	(1.0, 5.0)	323	323	323	0.4	19	94.7	9, 47, 24, 38, 30, 2, 37, 10, 45, 32, 21, 16, 36, 43, 31, 35, 34, 22, 7
	(1.5, 5.0)	322	322	322	0.4	19	84.2	9, 47, 12, 38, 24, 32, 37, 10, 45, 16, 21, 23, 36, 43, 31, 35, 34, 22, 7
	(2.0, 5.0)	329	329	329	0.4	20	85.0	9, 47, 24, 12, 38, 37, 10, 45, 11, 32, 23, 21, 16, 36, 43, 31, 35, 34, 22, 7
	(2.5, 5.0)	332	332	332	0.4	21	80.9	9, 47, 24, 12, 38, 37, 10, 45, 11, 32, 23, 21, 16, 36, 43, 31, 44, 35, 34, 22, 7
	(3.0, 5.0)	333	333	333	0.4	21	80.9	9, 47, 12, 38, 24, 32, 37, 10, 45, 41, 16, 21, 23, 36, 43, 31, 44, 35, 34, 22, 7
	(3.5, 5.0)	337	337	337	0.4	21	76.2	9, 47, 12, 38, 24, 32, 37, 10, 45, 11, 41, 16, 21, 23, 36, 43, 31, 35, 34, 22, 7
	(4.0, 5.0)	338	338	338	0.2	21	80.9	9, 47, 24, 38, 12, 32, 37, 10, 45, 41, 1, 16, 21, 26, 23, 43, 31, 35, 34, 22, 7
	(4.5, 5.0)	346	346	346	0.4	23	78.3	9, 47, 24, 38, 12, 32, 37, 10, 45, 41, 28, 1, 16, 21, 26, 36, 43, 31, 44, 35, 34, 22, 7
	(5.0, 5.0)	338	338	338	0.4	20	75	9, 47, 12, 38, 24, 32, 37, 10, 45, 41, 16, 21, 36, 43, 31, 42, 22, 34, 35, 7
	(1.0, 5.5)	317	317	317	0.4	20	90.0	9, 47, 24, 38, 12, 32, 37, 10, 45, 16, 21, 26, 36, 43, 31, 44, 35, 34, 22, 7
	(1.5, 5.5)	322	322	322	0.4	19	84.2	9, 47, 12, 38, 24, 32, 37, 10, 45, 16, 21, 23, 36, 43, 31, 35, 34, 22, 7
	(2.0, 5.5)	329	329	329	0.4	20	85.0	9, 47, 24, 12, 38, 37, 10, 45, 11, 32, 23, 21, 16, 36, 43, 31, 35, 34, 22, 7
	(2.5, 5.5)	332	332	332	0.4	21	80.9	9, 47, 24, 12, 38, 37, 10, 45, 11, 32, 23, 21, 16, 36, 43, 31, 44, 35, 34, 22, 7
	(3.0, 5.5)	333	333	333	0.4	21	80.9	9, 47, 12, 38, 24, 32, 37, 10, 45, 41, 16, 21, 23, 36, 43, 31, 44, 35, 34, 22, 7
	(3.5, 5.5)	337	337	337	0.4	21	76.2	9, 47, 12, 38, 24, 32, 37, 10, 45, 11, 41, 16, 21, 23, 36, 43, 31, 35, 34, 22, 7
	(4.0, 5.5)	337	337	337	0.4	22	81.8	9, 47, 24, 38, 12, 32, 37, 10, 45, 41, 1, 16, 21, 26, 36, 43, 31, 44, 35, 34, 22, 7
	(4.5, 5.5)	346	346	346	0.4	23	78.3	9, 47, 24, 38, 12, 32, 37, 10, 45, 41, 28, 1, 16, 21, 26, 36, 43, 31, 44, 35, 34, 22, 7
	(5.0, 5.5)	338	338	338	0.4	20	75.0	9, 47, 12, 38, 24, 32, 37, 10, 45, 41, 16, 21, 36, 43, 31, 42, 22, 34, 35, 7
	(1.0, 6.0)	323	323	323	0.4	19	94.7	9, 47, 24, 38, 30, 2, 37, 10, 45, 32, 21, 16, 36, 43, 31, 35, 34, 22, 7
	(1.5, 6.0)	322	322	322	0.4	19	84.2	9, 47, 24, 38, 12, 32, 37, 10, 45, 16, 21, 23, 36, 43, 31, 35, 34, 22, 7
	(2.0, 6.0)	329	329	329	0.4	20	85.0	9, 47, 24, 12, 38, 37, 10, 45, 11, 32, 23, 21, 16, 36, 43, 31, 35, 34, 22, 7
	(2.5, 6.0)	332	332	332	0.4	21	80.9	9, 47, 24, 12, 38, 37, 10, 45, 11, 32, 23, 21, 16, 36, 43, 31, 44, 35, 34, 22, 7
	(3.0, 6.0)	333	333	333	0.4	21	80.9	9, 47, 12, 38, 24, 32, 37, 10, 45, 41, 16, 21, 23, 36, 43, 31, 44, 35, 34, 22, 7
	(3.5, 6.0)	337	337	337	0.4	21	76.2	9, 47, 12, 38, 24, 32, 37, 10, 45, 11, 41, 16, 21, 23, 36, 43, 31, 35, 34, 22, 7
	(4.0, 6.0)	337	337	337	0.4	22	81.8	9, 47, 24, 38, 12, 32, 37, 10, 45, 41, 1, 16, 21, 26, 36, 43, 31, 44, 35, 34, 22, 7
	(4.5, 6.0)	346	346	346	0.4	23	78.3	9, 47, 24, 38, 12, 32, 37, 10, 45, 41, 28, 1, 16, 21, 26, 36, 43, 31, 44, 35, 34, 22, 7
	(5.0, 6.0)	338	338	338	0.4	20	75	9, 47, 12, 38, 24, 32, 37, 10, 45, 41, 16, 21, 36, 43, 31, 42, 22, 34, 35, 7
	(1.0, 6.5)	330	330	330	0.4	20	90.0	9, 47, 24, 38, 30, 2, 32, 37, 10, 45, 11, 36, 21, 16, 43, 31, 35, 34, 22, 7
	(1.5, 6.5)	333	333	333	0.4	21	85.7	9, 47, 24, 38, 30, 2, 32, 37, 10, 45, 11, 36, 21, 16, 43, 31, 44, 35, 34, 22, 7
	(2.0, 6.5)	336	336	336	0.4	21	76.2	9, 47, 24, 12, 38, 30, 2, 32, 37, 10, 45, 11, 21, 16, 36, 43, 31, 35, 34, 22, 7
	(2.5, 6.5)	338	338	338	0.4	21	80.9	9, 47, 24, 38, 30, 2, 32, 37, 10, 45, 11, 41, 36, 21, 16, 43, 31, 35, 34, 22, 7
	(3.0, 6.5)	341	341	341	0.5	22	81.8	9, 47, 24, 38, 30, 2, 32, 37, 10, 45, 11, 41, 36, 21, 16, 43, 31, 44, 35, 34, 22, 7
	(3.5, 6.5)	342	342	342	0.4	21	80.9	9, 47, 24, 38, 30, 2, 32, 37, 10, 45, 11, 36, 21, 16, 1, 43, 31, 35, 34, 22, 7
	(4.0, 6.5)	342	342	342	0.4	21	80.9	9, 47, 24, 38, 30, 2, 32, 37, 10, 45, 11, 36, 21, 16, 1, 43, 31, 35, 34, 22, 7
	(4.5, 6.5)	331	331	331	0.5	19	68.4	9, 47, 24, 38, 12, 32, 37, 10, 45, 21, 29, 8, 16, 43, 44, 35, 34, 22, 7
	(5.0, 6.5)	348	348	348	0.5	21	66.7	9, 47, 24, 12, 38, 30, 2, 32, 37, 45, 11, 10, 42, 21, 16, 43, 31, 35, 34, 22, 7
	(1.0, 7.0)	330	330	330	0.4	20	95.0	9, 24, 47, 38, 30, 2, 32, 37, 10, 45, 11, 21, 16, 36, 43, 31, 35, 34, 22, 7
	(1.5, 7.0)	333	333	333	0.4	21	95.2	9, 24, 47, 38, 30, 2, 32, 37, 10, 45, 11, 21, 16, 36, 43, 31, 44, 35, 34, 22, 7
	(2.0, 7.0)	337	337	337	0.4	21	85.7	9, 24, 47, 12, 38, 30, 2, 32, 37, 10, 45, 41, 21, 16, 36, 43, 31, 35, 34, 22, 7
	(2.5, 7.0)	336	336	336	0.4	22	77.3	9, 24, 47, 12, 38, 30, 2, 32, 11, 45, 10, 41, 21, 16, 36, 43, 31, 44, 35, 34, 22, 7
	(3.0, 7.0)	344	344	344	0.4	22	81.1	9, 24, 47, 12, 38, 30, 2, 32, 37, 10, 11, 45, 41, 21, 16, 36, 43, 31, 35, 34, 22, 7
	(3.5, 7.0)	337	337	337	0.2	21	76.2	9, 24, 12, 38, 47, 32, 37, 10, 45, 11, 2, 23, 21, 16, 36, 43, 31, 35, 34, 22, 7
	(4.0, 7.0)	329	329	329	0.4	20	70.0	24, 47, 30, 9, 10, 37, 45, 11, 32, 2, 23, 21, 16, 36, 43, 31, 35, 34, 22, 7
	(4.5, 7.0)	330	330	330	0.4	19	78.9	9, 24, 47, 12, 38, 32, 37, 45, 10, 21, 16, 36, 43, 31, 42, 22, 35, 34, 7
	(5.0, 7.0)	348	348	348	0.4	21	71.4	9, 24, 47, 12, 38, 30, 2, 32, 37, 11, 45, 10, 42, 21, 16, 43, 31, 35, 34, 22, 7

### Computational results on OPSTW instances

In this section we computationally test the increase of the profit due to soft time windows.

Table 4 presents the results obtained by HILS algorithm on OPSTW test instances. Column one (Instance) presents the instance over which the algorithm is tested. Column two corresponds to the maximum allowable time window violation and the maximum allowable waiting time at any customer. Columns three to six present the best, worst and average profit, over five runs, of HILS algorithm and the average computational time. Columns $N_R$ and %HTW present the number of routed customers on the average solution and the percentage of non-violated time windows on this solution respectively. The last column presents the sequence in which the customers are routed in the average solution.

The results show, as expected, that in all cases it is possible to increase the collected profit by allowing controlled violations of time windows. Allowing for example the maximum allowable violation of time window to 1 % of the maximum time duration and the maximum allowable waiting time at any customer to 7 % of the maximum time duration, results in solution with a profit of 330 and 95 % of non-violated time windows while the profit reported for hard time windows is 308. On the other hand, setting for example $P_{max}$ to 4.5 % and $W_{max}$ to 5 %, results in 23 routed customers while the number of routed customers reported for hard time windows is 21.

## Conclusions

In this paper we have introduced the orienteering problem with soft time windows (OPSTW). This routing problem can serve as a model for many practical applications for which travel times cannot be accurately known or when hard time windows are not required. Computational results on OPSTW show that our hybrid algorithm is able to achieve solutions that increase the total collected profit without hurting customers’ satisfaction significantly. On OPTW test instances our hybrid algorithm is able to achieve promising solutions. In our test experiments, instances with tight time windows are solved better than that with broader time windows. A 2-Opt or 3-Opt procedure may reduce this gap by decreasing the time duration of the route and inserting other possible unrouted customers. Since the chosen acceptance criterion has a critical influence on the balance between intensification and diversification of the search, a possible improvement of the algorithm could involve also considering worst solutions during the search. One could work with a simulated annealing acceptance criterion.
